# Supplementary material for: Histone-methyltransferase KMT2D deficiency impairs the Fanconi anemia/BRCA pathway upon glycolytic inhibition in squamous cell carcinoma
Source: Nat Commun. 2024 Aug 8;15:6755. doi: 10.1038/s41467-024-50861-5 (PMC11310337; doi:10.1038/s41467-024-50861-5)
Supplement: Supplementary file 1 — Supplementary Information [file 41467_2024_50861_MOESM1_ESM.pdf]

SUPPLEMENTARY INFORMATION

For paper entitled "**Histone-methyltransferase KMT2D Deficiency Impairs the Fanconi Anemia/BRCA Pathway upon Glycolytic Inhibition in Squamous Cell Carcinoma**"

Wei Liu[1,2,4], Hongchao Cao[1,2,4], Jing Wang[1,2], Areeg Elmusrati[1,2], Bing Han[1,2], Wei Chen[1,2], Ping Zhou[1,2], Xiyao Li[1,2], Stephen Keysar[3], Antonio Jimeno[3], Cun-Yu Wang[1,2]*

[1]Jonsson Comprehensive Cancer Center, University of California, Los Angeles, Los Angeles, CA, USA.

[2]Laboratory of Molecular Signaling, Division of Oral and Systemic Health Sciences, School of Dentistry, University of California, Los Angeles, Los Angeles, CA, USA.

[3]Division of Medical Oncology, Department of Medicine, University of Colorado Anschutz Medical Campus, Aurora, CO, USA.

[4]These authors contribute equally to this work.

*Corresponding author. Email: cwang@dentistry.ucla.edu

Supplementary Table 1. Oligonucleotides for sgRNA, qRT-PCR, and ChIP-qPCR.

Oligonucleotides		
Primers for qRT-PCR		
Gene	Forward (5'-3')	Reverse (5'-3')
Human ATR	CTGACTCTCAGCCAACCTCC	ACAACTGCCTTTGGCCTCAT
Human B2M	TCCATCCGACATTGAAGTT	GGCAGGCATACTCATCTTT
Human BRCA1	TCAAGGAACCTGTCTCCACA	AAAGGACACTGTGAAGGCCC
Human BRCA2	AGAGAAGCTGCAAGTCATGGA	TCCAATGTGGTCTTTGCAGC
Human EME1	ACAGTACTGGTGTTGCTCCG	TGACAGAGCTTTCCCTGCTG
Human ENO1	CTCCCAACATCCTGGAGAATAAA	CATGCCGATGACCACCTTAT
Human FAN1	CATTCCTTTGGAGCAGGGGT	CGGTTTTCAGCACCACAAGG
Human FANCD2	AAACAGAATGAAGCCAGCAGC	TGATTCACTTCTGTCTGCCGC
Human FANCI	ACTCAGGGCCTCGTAGAACT	TGCATGCTGGTTTGGCATTC
Human FANCM	TGCAATACGCTGGTGTGTCT	TGCTGTGTCACCAAGGGTTT
Human GCLC	CGGAGGAACAATGTCCGAGT	CCTTCCACTGGGTTGGGTTT
Human HK2	ATGGAGAAAGGGCTTGGAGC	CCAAAGCACACGGAAGTTGG
Human HKDC1	CAAGGACATGGACGTGGACA	TAGGGGTCGTCATAGGCACA
Human KMT2A	CTCCCACCTTCCCTGGAGTA	GTGGCTTGCTGAAACGTAGC
Human KMT2B	GTCGCAAGCATAAGACGACC	ACCATCCGTTCTGTGCCTTC
Human KMT2C	AACCAATGGCTGGAAATGCAA	TGCCACTGAGAACTAGACCG
Human KMT2D	CCCGGTGTGTAGCAGATTTT	TTCCTTGGGTTGCATAGAGG
Human LDHB	CCAGGATTCATCCCGTGTCA	GTCCCACAGGGTATCTGCAC
Human PGK1	GCCAAGTCGGTAGTCCTTATG	CCCAGCAGAGATTTGAGTTCTA
Human POLH	GTGGGAGCAGTGATTGTGGA	GAAGAGCTGTGGGACTGACC
Human POLQ	CCGAGGGTATCTGCTGGAAC	AGGGTAGCACTCATGCCAAC
Human REV3L	AGTCCTGATGAGGCTCTGGT	AAGGAGCGACATGGTGATGG
Human SLC16A1	TTTCTTTGCGGCTTCCGTTG	CTCTGGGGTCCAACAAGGTC
Human SLX4	GAAGAAACCTCCGTCTGGCA	CGTCTGGGTGTTTTGTGCTG
Human TOP3A	AGTTTAAGCGCGGTAGCCTT	CAGGTCCAGGATCTCCCTCA
Mouse Kmt2d	GCTATCACCCGTACTGTGTCAACA	CACACACGATACACTCCACACAA
Mouse B2m	CTCGGTGACCCTGGTCTTTC	GGATTTCAATGTGAGGCGGG
sgRNA-targeted sequence		
sgRNA name	Sequence (5'-3')	Edited cell line
sgLacZ	TGCGAATACGCCCACGCGAT	SCC23 and SCC1
sgKMT2D-1	ATTGATGCTACGTTGACCGG	SCC23 KO1
sgKMT2D-2	GGTGGAAATTCCCGCCAACG	SCC23 KO2 and SCC1 KO1

sgKMT2D-3	CCAAGTCATCTCAGTACCGG	SCC1 KO2
ChIP-qPCR primers for anti-KMT2D		
Gene	Forward (5'-3')	Reverse (5'-3')
Human ATR	CGTGGTTGACTAGTGCCTCG	TGAAACCCAAGCCGGAATCA
Human FANCM	CGGGTTGAAAAACCTACCGC	CAGAGGTCGCTTCACAAGGT
Human REV3L	CCACGGTGGTGCTTTTATGC	GGCCTCCTCGTGTTTTTCAC
Human TOP3A	ATGATCTTTCCTGTCGCCCG	CACACAGAGGACTTTCCGCA
ChIP-qPCR primers for anti-H3K4me1		
Gene	Forward (5'-3')	Reverse (5'-3')
Human ATR	CAGGCTCATTGTGAAGCTGA	CGGAGGAAGACAGAAGAGGG
Human FANCM	TGACAGCTTACACCGCATTG	GAAATGGTGGTGTGTGCCTC
Human REV3L	AGGATATACTGTGGCATTTCTGT	TGACTTGTGTGGAGGAGAAAA
Human TOP3A	AATAGGTTCCAGCGGCTCAG	AAGTGCCAGCCTGTTCCATT
ChIP-qPCR primers for Negative		
Gene	Forward (5'-3')	Reverse (5'-3')
Human ATR	TGGCTGATCAAGCTGCATGT	AGCTCCAGGACTGAACTCCA
Human FANCM	AGGGCTTTGCTCACATTGGA	TGCCTCCAAACAAATCCCCA
Human REV3L	AACGGGAACATGGATCCCAC	GGCCCCCAAAGCTAGTTAGT
Human TOP3A	TGTGCAGGAGATGCTAACAC	CTTTACAAAACTCGGCCTGACC

Supplementary Table 2. Chemicals and reagents.

Chemicals and reagents		
Name	Vendor	Catalog
DMEM	Thermo Fisher Scientific	Cat#11995065
DMEM, no glucose	Thermo Fisher Scientific	Cat#11966025
Fetal Bovine Serum	Thermo Fisher Scientific	Cat#26140079
Penicillin-Streptomycin (10,000 U/mL)	Thermo Fisher Scientific	Cat#15140122
Trypsin-EDTA	Thermo Fisher Scientific	Cat#R001100
Lipofectamine 2000	Thermo Fisher Scientific	Cat#11668019
TRIzol Reagent	Thermo Fisher Scientific	Cat#15596026
M-MuLV Reverse Transcriptase	New England Biolabs	Cat#M0253L
RNase Inhibitor	New England Biolabs	Cat#0307L
Random Hexamers	Thermo Fisher Scientific	Cat#N8080127
dNTP	Promega	Cat#U1515
Hot Start DNA Polymerase	New England Biolabs	Cat#M0481L
Tamoxifen	Sigma-Aldrich	Cat#5648
4-Nitroquinoline N-oxide (4NQO)	Santa Cruz	Cat#sc-256815
ProLong Diamond Antifade Mountant with DAPI	Thermo Fisher Scientific	Cat#P36962
RIPA Buffer	Sigma-Aldrich	Cat#R0278
Fisherbrand Superfrost Plus Microscope Slides	Thermo Fisher Scientific	Cat#12-550-15
Halt Protease Inhibitor Cocktail	Thermo Fisher Scientific	Cat#78430
Phosphatase inhibitors	Sigma-Aldrich	Cat#4906845001
MTT	Thermo Fisher Scientific	Cat#M6494
2-Deoxy-D-glucose	MedChem Express	Cat#HY-13966
Mitomycin C	Selleck Chemicals	Cat#S8146
Cisplatin	Selleck Chemicals	Cat#S1166
Olaparib	MedChem Express	Cat#HY-10162
Compound C	EMD Millipore	Cat#171261

Supplementary Figure 1

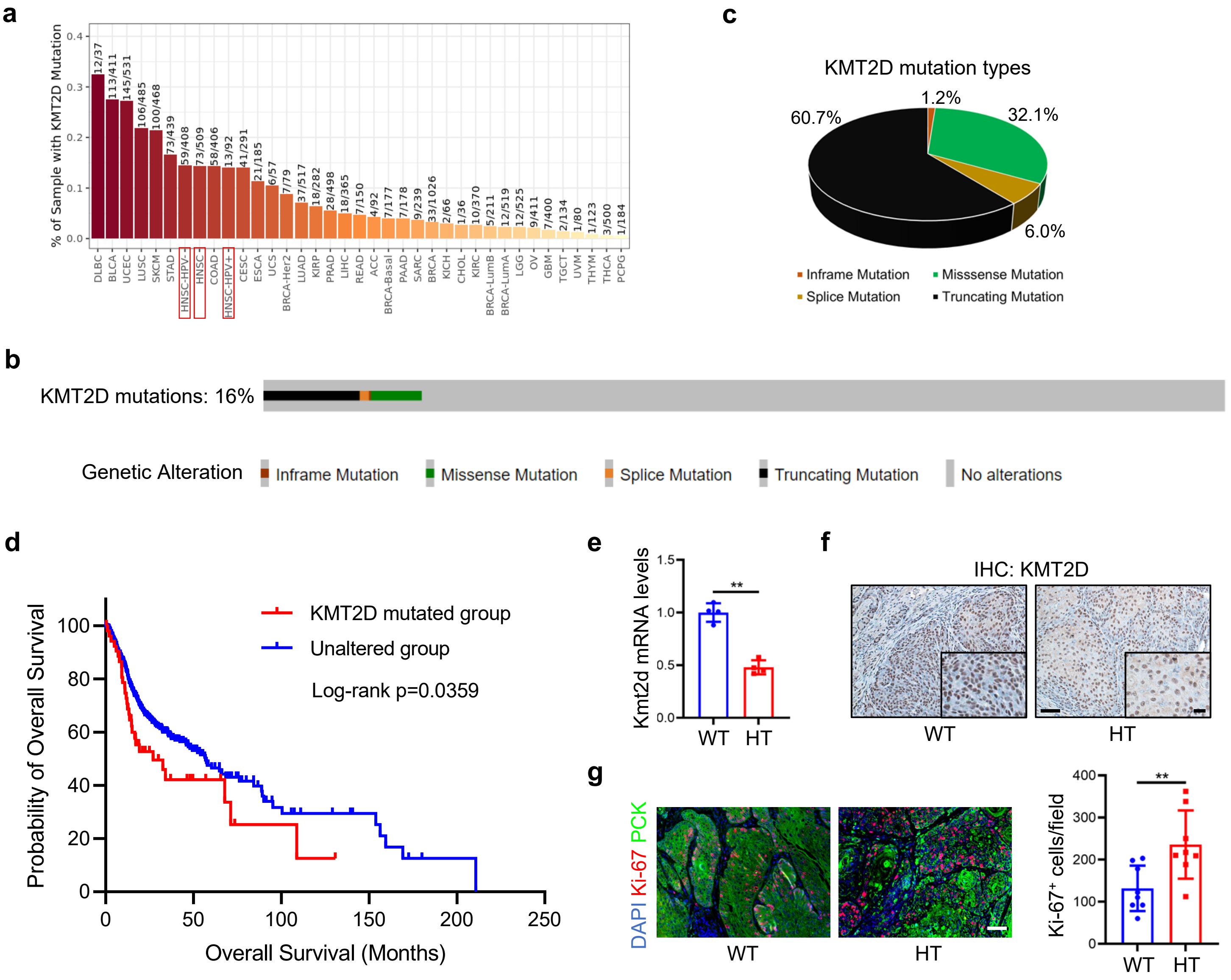

Supplementary Fig. 1: KMT2D is highly mutated in HNSCC and epithelial heterozygous deletion of *Kmt2d* increases mouse HNSCC proliferation. a, Mutation rates of KMT2D in different types of tumor using TIMER 2.0. **b,** KMT2D mutation profile in Head and Neck Squamous Cell Carcinoma (TCGA, Firehose Legacy, n=512) from cBioPortal. **c,** The percentage of truncating (loss-of-function) mutations in total mutations in HNSCC (TCGA, Firehose Legacy, n=512) from cBioPortal. **d,** Kaplan-Meier curve analysis of the overall survival (OS) of HNSCC patients stratified into KMT2D mutated and unaltered groups (TCGA, Firehose Legacy, n=53 for KMT2D mutated group with loss-of-function mutations and n=442 for unaltered group). P values were calculated by log-rank test. **e,** mRNA levels of *Kmt2d* in tongue epithelia from *Kmt2d*-WT and *Kmt2d*-HT mice two weeks after Tam injection by qRT-PCR. Values are mean ± SD. n=4:4. **p < 0.01 by unpaired two-tailed Student's t test. **f,** Representative IHC staining of KMT2D in *Kmt2d*-WT and *Kmt2d*-HT HNSCC of mice. n=12:12. Enlarged images are in the inserts. Scale bar, 50 µm. **g,** Representative IF staining and quantification of Ki-67 positive cells. Scale bar, 50 µm. Values are mean ± SD. n=8:8. **p < 0.01 by unpaired two-tailed Student's t test.

Supplementary Figure 2

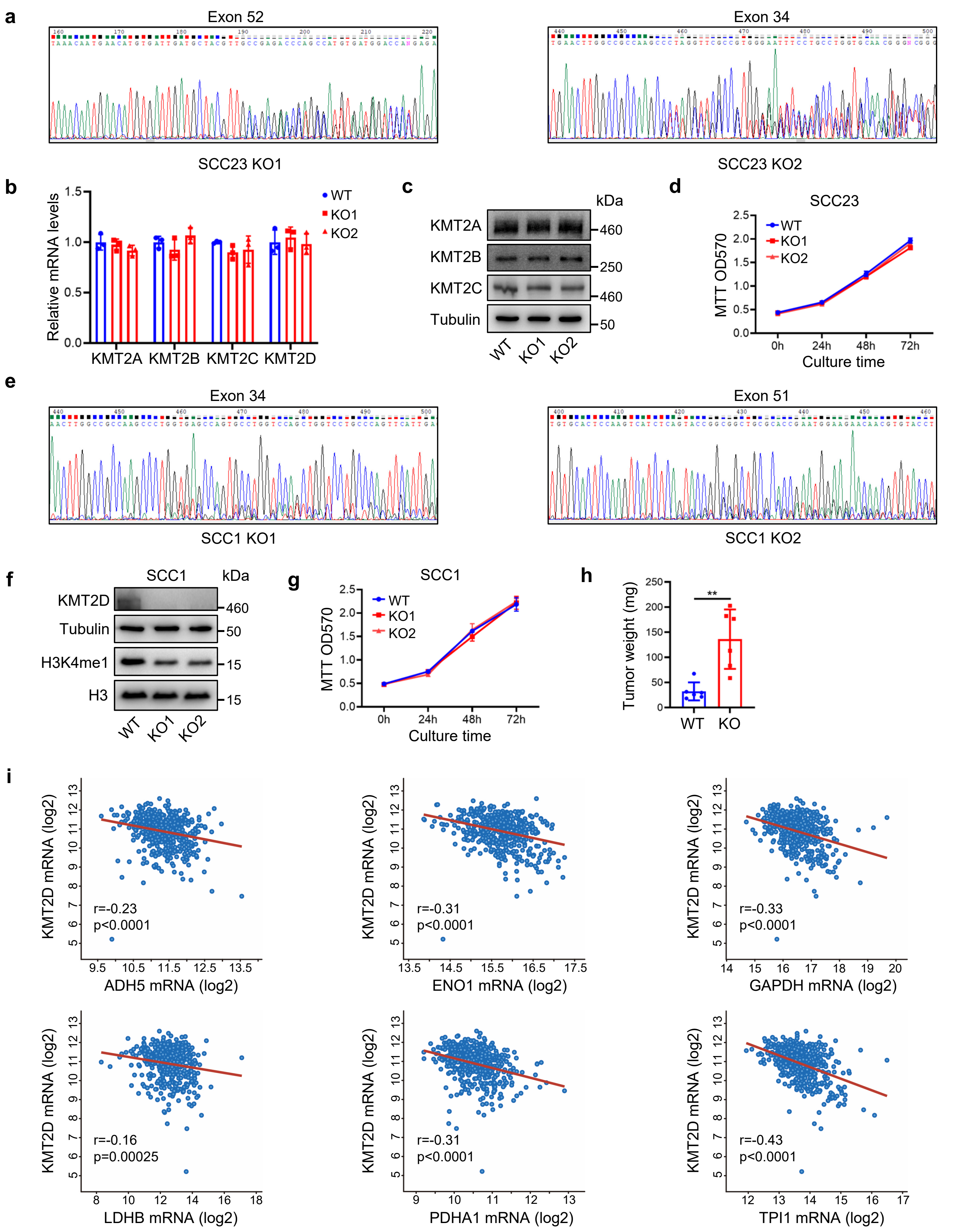

Supplementary Fig. 2: *KMT2D* expression levels are negatively correlated with the expression of several glycolytic genes. a, Sanger sequencing analysis of DNA fragments from KMT2D-KO SCC23 cells. **b,** mRNA levels of *KMT2A*, *KMT2B*, *KMT2C*, and *KMT2D* from KMT2D-WT and KMT2D-KO SCC23 cells by qRT-PCR. Values are mean ± SD from three independent experiments. **c,** Protein levels of KMT2A, KMT2B, and KMT2C from KMT2D-WT and KMT2D-KO SCC23 cells by western blot. n = 3 independent experiments. **d,** Growth curves of KMT2D-WT and KMT2D-KO SCC23 cells performed by MTT assay. **e,** Sanger sequencing analysis of DNA fragments from KMT2D-KO SCC1 cells. **f,** Protein levels of KMT2D and H3K4me1 in KMT2D-WT and KMT2D-KO SCC1 cells by western blot. n = 3 independent experiments. **g,** Growth curves of KMT2D-WT and KMT2D-KO SCC1 cells performed by MTT assay. **h,** Xenograft tumor weight of KMT2D-WT and KMT2D-KO SCC1 cells. Values are mean ± SD. n=6:6. **p < 0.01 by unpaired two-tailed Student's t test. **i,** Inverse correlations of *KMT2D* mRNA levels with *ADH5*, *ENO1*, *GAPDH*, *LDHB*, *PDHA1*, and *TPI1* mRNA levels in human HNSCC Firehose Legacy TCGA dataset (n = 496) from cBioPortal. r, Pearson's correlation coefficient.

Supplementary Figure 3

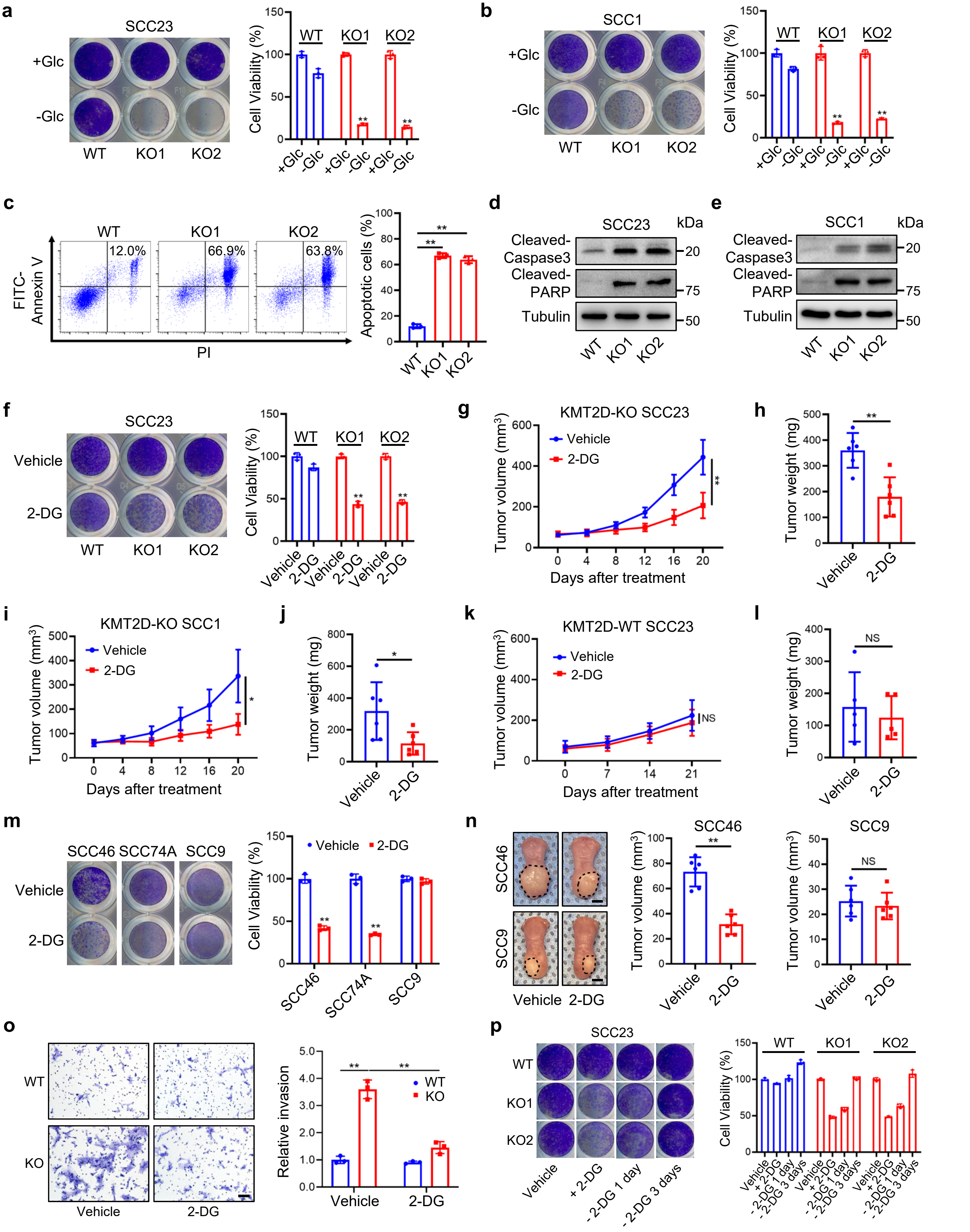

Supplementary Fig. 3: Inhibition of glycolysis increases HNSCC apoptosis and reduces tumor growth of KMT2D-deficient HNSCC. a,b, Crystal violet staining and cell viability of KMT2D-WT and KMT2D-KO SCC23 (**a**) and SCC1 (**b**) cells cultured under glucose-sufficient (+Glc) or glucose-deprived (-Glc) conditions. Values are mean ± SD. **p < 0.01 vs WT by one-way ANOVA in -Glc condition. **c,** Representative scatter plots and quantification of apoptotic SCC23 cells following glucose deprivation. Values are mean ± SD from three independent experiments. **p < 0.01 by one-way ANOVA. **d,e,** Protein levels of cleaved Caspase-3 and cleaved PARP from SCC23 (**d**) and SCC1 (**e**) cells after glucose deprivation by western blot. n = 3 independent experiments. **f,** Crystal violet staining and cell viability of SCC23 cells treated with 1mM 2-DG for 3 days. **p < 0.01 vs WT by one-way ANOVA in 2-DG treated condition. **g,h,** Xenografted tumor growth (**g**) and weight (**h**) of KMT2D-KO SCC23 cells. Values are mean ± SD. n=6:6. **p < 0.01 by two-way ANOVA (**g**). **p < 0.01 by Student's t test (**h**). **i,j,** Xenografted tumor growth (**i**) and weight (**j**) of KMT2D-KO SCC1 cells treated with 2-DG or vehicle. n=6:6. *p < 0.05 by two-way ANOVA (**i**). *p < 0.05 by Student's t test (**j**). **k,l,** Xenografted tumor growth (**k**) and weight (**l**) of KMT2D-WT SCC23 cells treated with 2-DG or vehicle. Values are mean ± SD. n=5:5. NS, not significant by two-way ANOVA (**k**) or by Student's t test (**l**). **m,** Crystal violet staining and cell viability of SCC46 and SCC74A cells with KMT2D-inactivating mutations and SCC9 cells bearing KMT2D-WT. Values are mean ± SD. **p < 0.01 by unpaired two-tailed Student's t test. **n,** Volumes of SCC46 and SCC9 orthotopic xenografts. Scale bar, 2 mm. n=6:6. **p < 0.01 by Student's t test. **o,** Invasion abilities of KMT2D-WT and KMT2D-KO SCC23 cells determined by transwell assay. Values are mean ± SD from three independent experiments. **p < 0.01 by two-way ANOVA. **p,** Crystal violet staining and cell viability of KMT2D-WT and KMT2D-KO SCC23 cells treated with 1mM 2-DG for 3 days, and then 2-DG was removed for another 1 day or 3 days as indicated.

Supplementary Figure 4

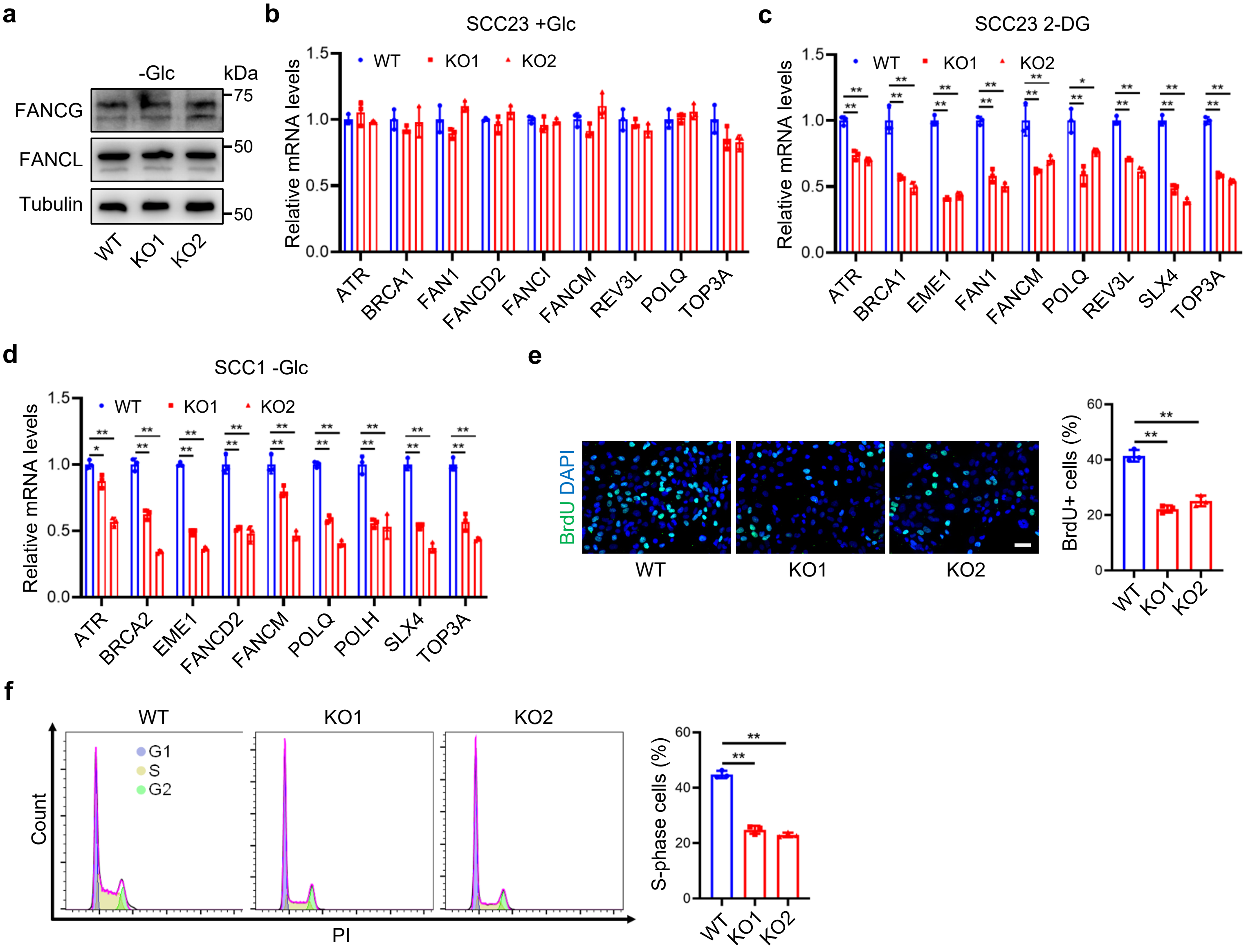

Supplementary Fig. 4: KMT2D loss downregulates FA genes in HNSCC upon glycolytic inhibition. **a,** Protein levels of FANCG and FANCL in KMT2D-WT and KMT2D-KO SCC23 cells under glucose deprivation. n = 3 independent experiments. **b,** mRNA levels of FA genes in KMT2D-WT and KMT2D-KO SCC23 cells cultured under glucose-sufficient conditions. Values are mean ± SD from three independent experiments. **c,** mRNA levels of FA genes in KMT2D-WT and KMT2D-KO SCC23 cells upon 2-DG treatment. Values are mean ± SD from three independent experiments. *p < 0.05, **p < 0.01 by one-way ANOVA. **d,** mRNA levels of FA genes in KMT2D-WT and KMT2D-KO SCC1 cells after glucose deprivation. Values are mean ± SD from three independent experiments. *p < 0.05, **p < 0.01 by one-way ANOVA. **e,** Representative image of BrdU incorporation in KMT2D-WT and KMT2D-KO SCC23 cells after 2-DG treatment. Scale bar, 50 μm. Values are mean ± SD. n=3 per group. **p < 0.01 by one-way ANOVA. **f,** Representative cell cycle analysis determined by PI staining and quantification of S-phase cells in KMT2D-WT and KMT2D-KO SCC23 cells after 2-DG treatment. Values are mean ± SD. n=3 per group. **p < 0.01 by one-way ANOVA.

Supplementary Figure 5

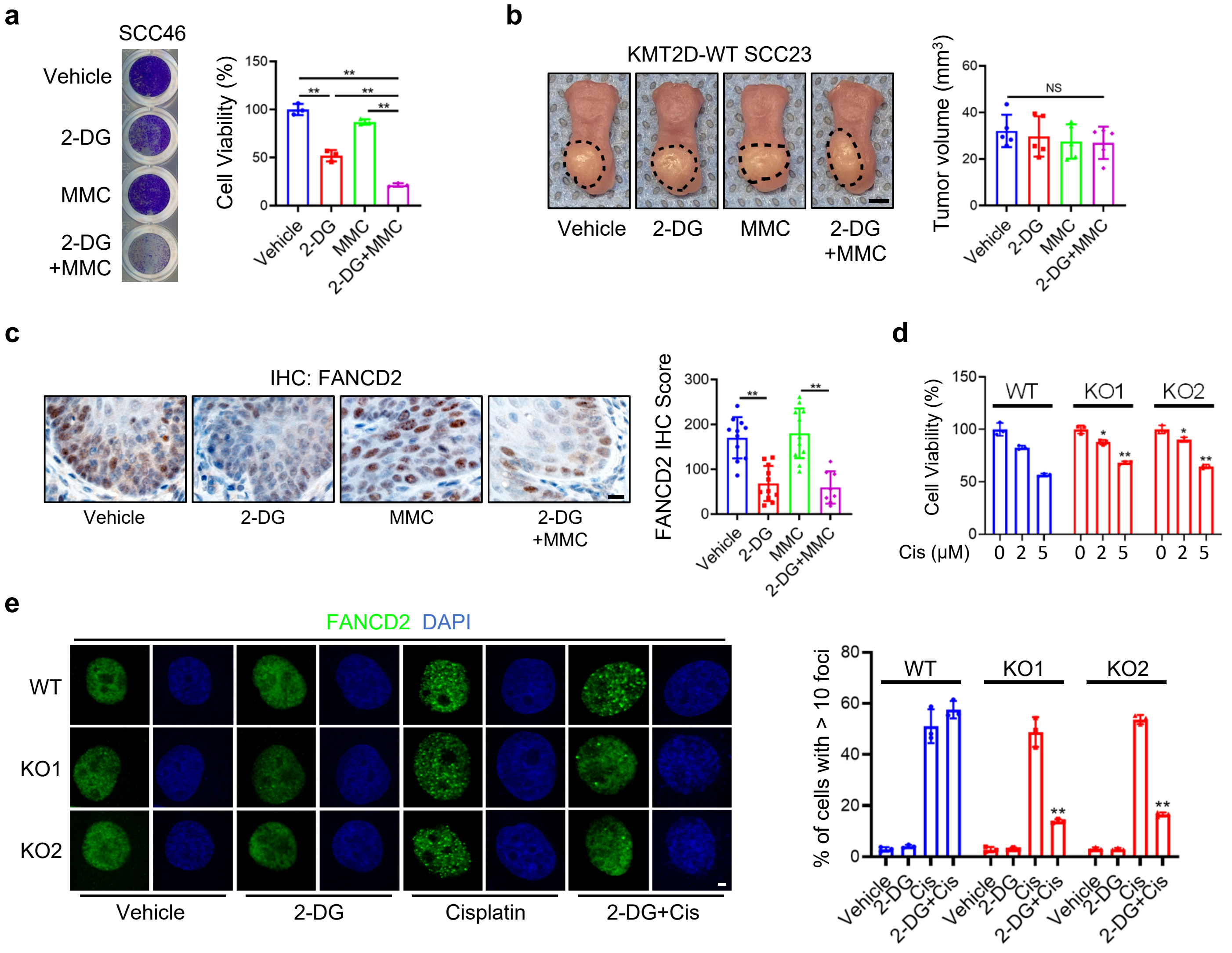

Supplementary Fig. 5: KMT2D-deficient HNSCC is hypersensitive to DNA cross-linking agents upon glycolytic inhibition. a, Crystal violet staining and cell viability of SCC46 cells treated with 2-DG, MMC, or 2-DG plus MMC. Values are mean ± SD from three independent experiments. **p < 0.01 by one-way ANOVA. **b,** Tumor volume of KMT2D-WT SCC23 orthotopic xenografts treated with 2-DG, MMC, or 2-DG plus MMC in nude mice. Scale bar, 2 mm. Values are mean ± SD. n=5 per group. NS, not significant. **c,** IHC staining and quantification of FANCD2 in mouse *Kmt2d*-HT HNSCC. Scale bar, 10 μm. Values are mean ± SD. **p < 0.01 by one-way ANOVA. **d,** Cell viability of KMT2D-WT and KMT2D-KO SCC23 cells treated with different concentrations of cisplatin. Values are mean ± SD from three independent experiments. *p < 0.05, **p < 0.01 vs WT in the corresponding treated conditions by one-way ANOVA. **e,** IF staining of FANCD2 and quantification of FANCD2 foci in KMT2D-WT and KMT2D-KO SCC23 cells treated with 2-DG, cisplatin, or 2-DG plus cisplatin for 24h. Scale bar, 2 μm. Values are mean ± SD from three independent experiments. **p < 0.01 vs WT in 2-DG plus cisplatin group by one-way ANOVA.

Supplementary Figure 6

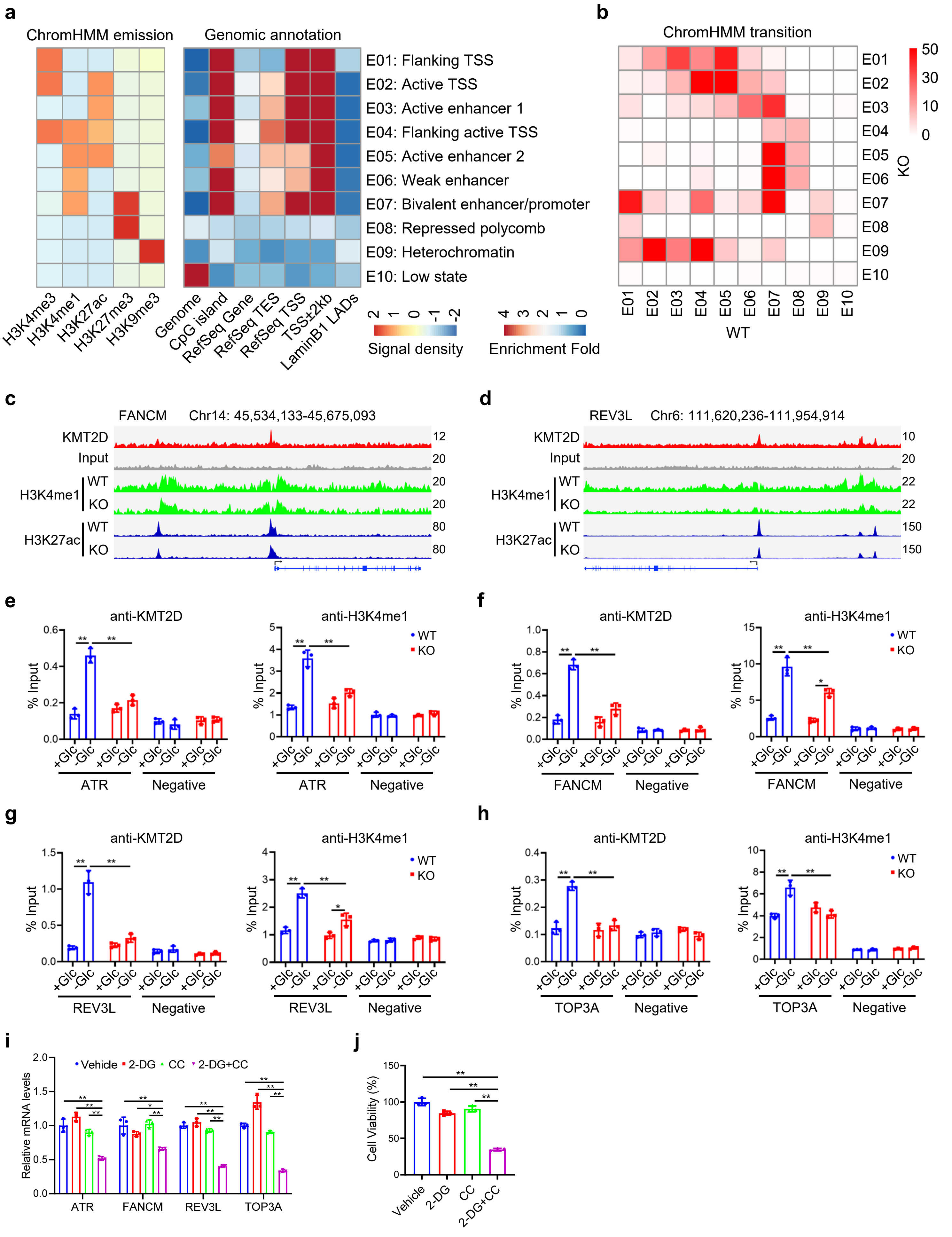

Supplementary Fig. 6: KMT2D epigenetically regulates FA genes upon glycolytic inhibition.

a, Chromatin states were defined by enrichment of histone modifications using the ChromHMM algorithm in KMT2D-WT and KMT2D-KO SCC23 cells after glucose deprivation for 6 hours. Heatmap of histone modification emission probabilities in chromatin states (left). Genomic annotation enrichments for each chromatin state (right). **b,** Heatmap of the relative abundance changes of defined 10 chromatin states between KMT2D-WT and KMT2D-KO SCC23 cells under glucose deprivation. **c,d,** ChIP-seq binding signals of KMT2D in KMT2D-WT SCC23 cells and H3K4me1, H3K27ac for *FANCM* (**c**) and *REV3L* (**d**) in KMT2D-WT and KMT2D-KO SCC23 cells under glucose deprivation. **e-h**, ChIP-qPCR analysis of KMT2D and H3K4me1 at *ATR* (**e**), *FANCM* (**f**), *REV3L* (**g**), and *TOP3A* (**h**) locus in KMT2D-WT and KMT2D-KO SCC23 cells under glucose-sufficient (+Glc) or glucose-deprived (-Glc) conditions. Values are mean ± SD from three independent experiments. *$p < 0.05$, **$p < 0.01$ by two-way ANOVA with Bonferroni correction. **i,** mRNA levels of *ATR*, *FANCM*, *REV3L* and *TOP3A* in SCC23 cells treated with 2-DG, Compound C, or 2-DG plus Compound C. Values are mean ± SD from three independent experiments. *$p < 0.05$, **$p < 0.01$ by one-way ANOVA. **j,** Cell viability of SCC23 cells treated with 2-DG, Compound C, or 2-DG plus Compound C. Values are mean ± SD from three independent experiments. **$p < 0.01$ by one-way ANOVA.

Supplementary Figure 7

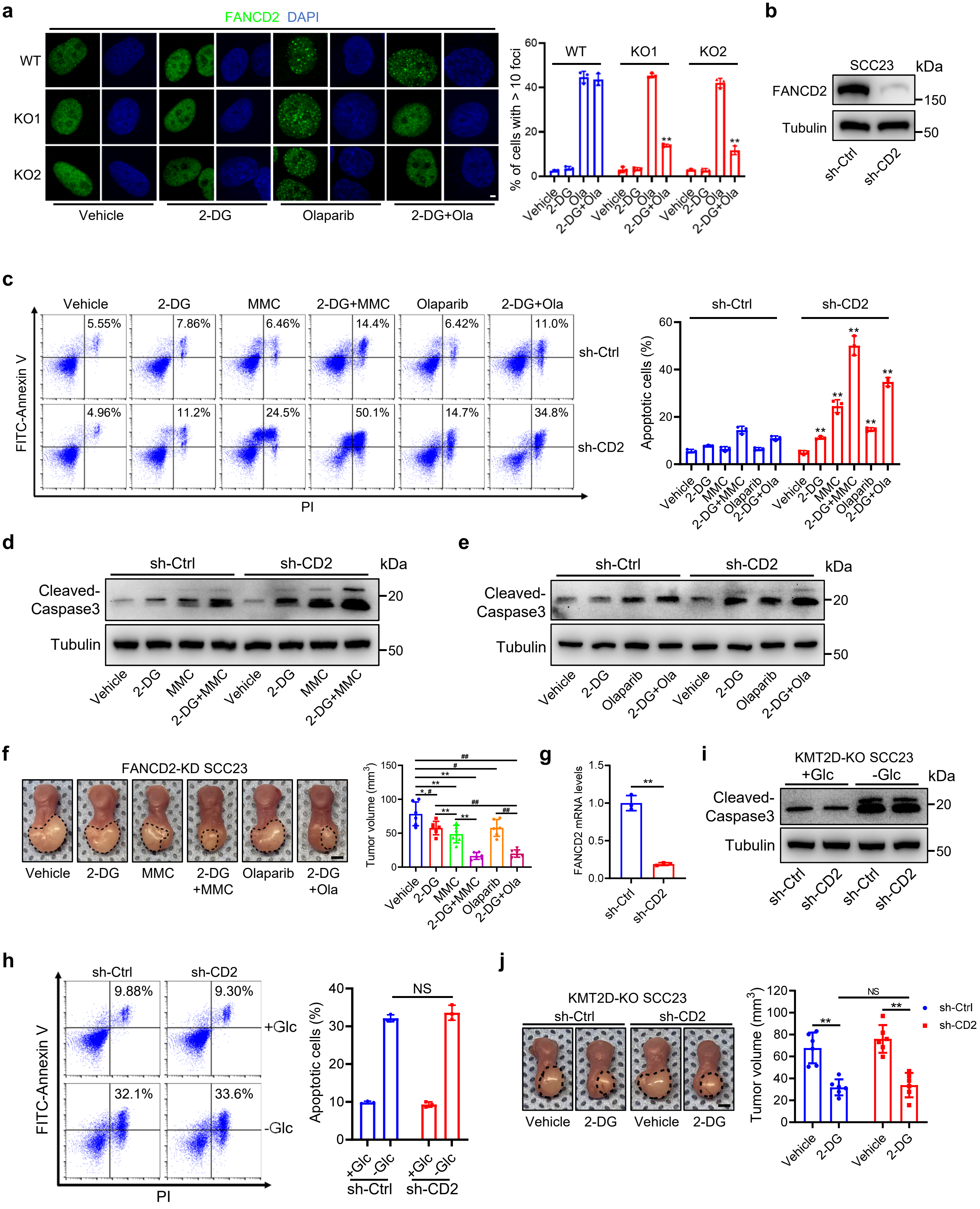

Supplementary Fig. 7: The hypersensitive KMT2D-deficient HNSCC to DNA damaging agents upon glycolytic inhibition depends on FA pathway impairment. a, IF staining of FANCD2 and quantification of FANCD2 foci in KMT2D-WT and KMT2D-KO SCC23 cells treated with 2-DG, olaparib, or 2-DG plus olaparib. Scale bar, 2 μm. Values are mean ± SD from three independent experiments. **p < 0.01 vs WT in 2-DG plus olaparib group by one-way ANOVA. **b,** Protein levels of FANCD2 in sh-Ctrl and sh-FANCD2 SCC23 cells by western blot. n = 3 independent experiments. **c,** Representative scatter plots and quantification of apoptotic sh-Ctrl and sh-FANCD2 SCC23 cells treated with 2-DG, MMC, 2-DG plus MMC, olaparib, or 2-DG plus olaparib. Values are mean ± SD from three independent experiments. **p < 0.01 vs sh-Ctrl in the corresponding treated conditions by unpaired two-tailed Student's t test. **d,e,** Protein levels of cleaved Caspase-3 treated with 2-DG, MMC, or 2-DG plus MMC (**d**) or treated with 2-DG, olaparib, or 2-DG plus olaparib (**e**). n = 3 independent experiments. **f,** Volume of FANCD2 knockdown SCC23 orthotopic xenografts treated with 2-DG, MMC, 2-DG plus MMC, olaparib, or 2-DG plus olaparib in nude mice. Scale bar, 2 mm. Values are mean ± SD. n=6 per group. *p < 0.05, **p < 0.01 by one-way ANOVA. **g,** Relative mRNA levels of FANCD2 after shRNA knockdown in KMT2D-KO SCC23 cells. **p < 0.01 by unpaired two-tailed Student's t test. **h,** Representative scatter plots and quantification of apoptotic FANCD2-KD, KMT2D-KO SCC23 cells and KMT2D-KO SCC23 cells following glucose deprivation. Values are mean ± SD from three independent experiments. NS, not significant by two-way ANOVA with Bonferroni correction. **i,** Protein levels of cleaved Caspase-3 from FANCD2-KD, KMT2D-KO SCC23 cells and KMT2D-KO SCC23 cells after glucose deprivation by western blot. n = 3 independent experiments. **j,** Volume of FANCD2-KD and KMT2D-KO SCC23 orthotopic xenografts treated with 2-DG in nude mice. Scale bar, 2 mm. Values are mean ± SD. n=6 per group. **p < 0.01 by two-way ANOVA.

Supplementary Figure 8

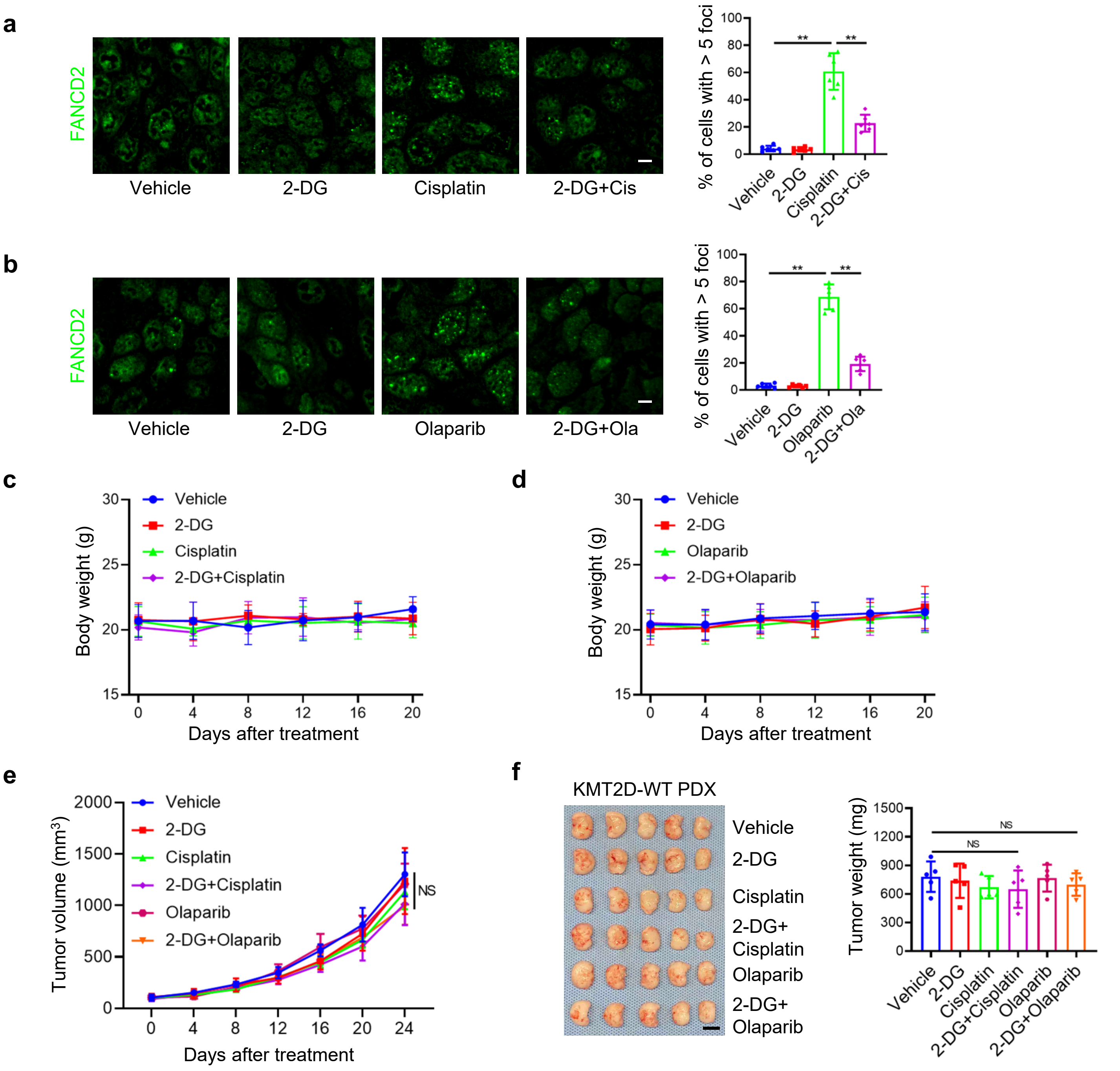

Supplementary Fig. 8: KMT2D-deficient HNSCC is hypersensitive to DNA cross-linking agents plus 2-DG or PARP inhibitors plus 2-DG. a,b, IF staining of FANCD2 and quantification of FANCD2 foci in KMT2D-mutant PDX treated with 2-DG, cisplatin, or 2-DG plus cisplatin (**a**) or treated with 2-DG, olaparib, or 2-DG plus olaparib (**b**). Scale bar, 5 μm. Values are mean ± SD. n=6 per group. **p < 0.01 by one-way ANOVA. **c,d,** Body weights of NSG mice bearing KMT2D-mutant PDX treated with 2-DG, cisplatin, or 2-DG plus cisplatin (**c**) or treated with 2-DG, olaparib, or 2-DG plus olaparib (**d**). **e,** Tumor volume of KMT2D-WT PDX following treatments with 2-DG, cisplatin, olaparib, and the combinations. Values are mean ± SD. n=5 per group. NS, not significant by two-way ANOVA with Bonferroni correction. **f,** Tumor weight of KMT2D-WT PDX treated with 2-DG, cisplatin, olaparib, and the combinations. Scale bar, 1 cm. NS, not significant by one-way ANOVA.

Supplementary Figure 9

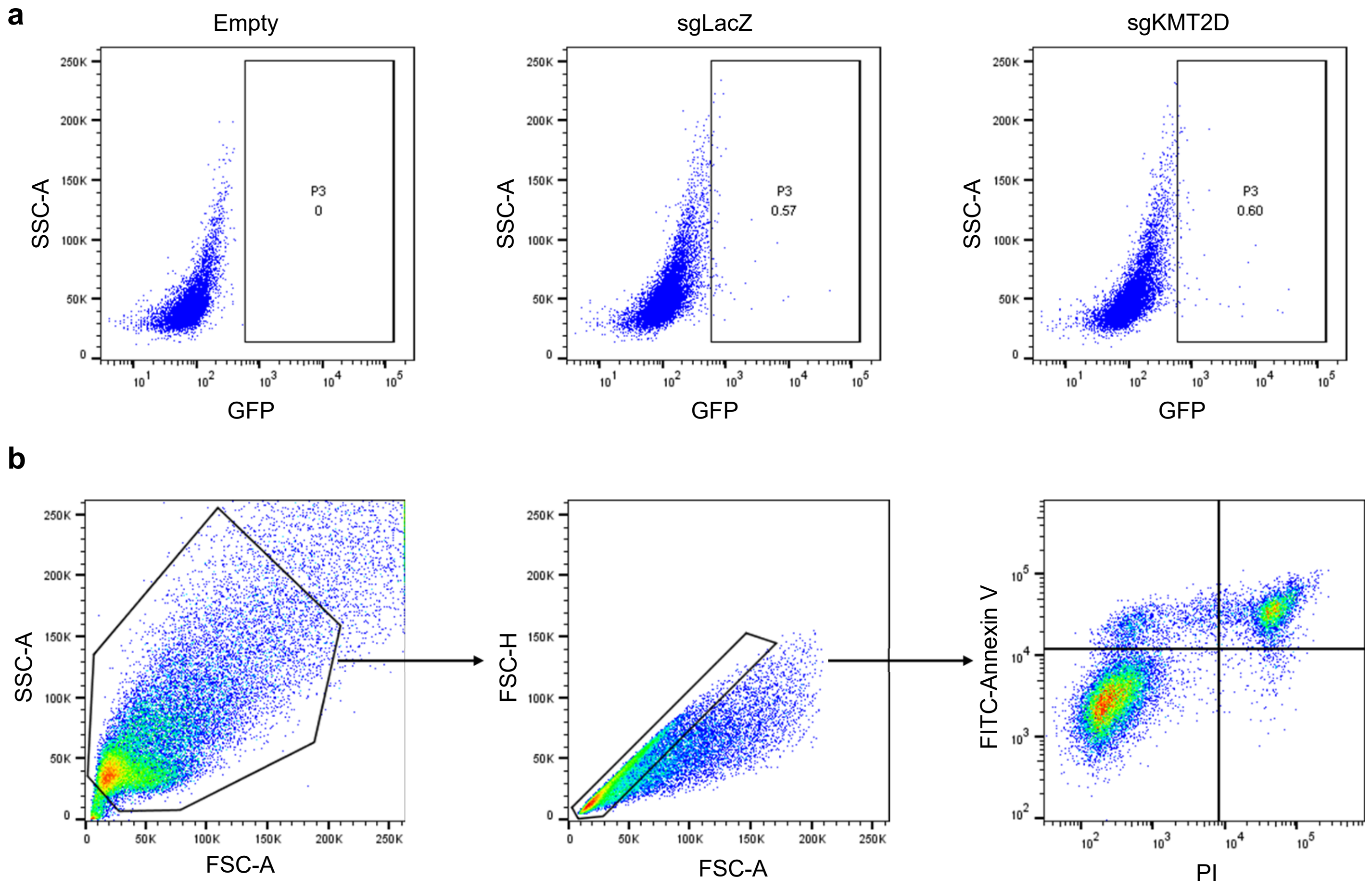

Supplementary Fig. 9: The gating strategies for the GFP sorting and FITC Annexin V apoptosis analysis. a, Sorting gate for GFP positive cells transfected with pSpCas9(BB)-2A-GFP-sgLacZ and pSpCas9(BB)-2A-GFP-sgKMT2D The un-transfected cells were used to set negative gate. **b**, SCC23 cells were gated on forward (FSC) vs side scatter (SSC) to select cell population (left). Selected cells were then gated on FSC-H vs FSC-A to generate the singlets gate (middle). Single cell sub-populations were analyzed on the Annexin V-FITC versus PI scatter for apoptosis (right). The gating strategies are for Fig.4a, Supplementary Fig. 3c, and Supplementary Fig. 7c,h.
