# Supplementary material for: Histone-methyltransferase KMT2D deficiency impairs the Fanconi anemia/BRCA pathway upon glycolytic inhibition in squamous cell carcinoma
Source: Nat Commun. 2024 Aug 8;15:6755. doi: 10.1038/s41467-024-50861-5 (PMC11310337; doi:10.1038/s41467-024-50861-5)
Supplement: Supplementary file 3 — Reporting Summary [file 41467_2024_50861_MOESM3_ESM.pdf]

nature portfolio

Corresponding author(s): Cun-Yu Wang

Last updated by author(s): Jul 1, 2024

Reporting Summary

Nature Portfolio wishes to improve the reproducibility of the work that we publish. This form provides structure for consistency and transparency in reporting. For further information on Nature Portfolio policies, see our Editorial Policies and the Editorial Policy Checklist.

Statistics

For all statistical analyses, confirm that the following items are present in the figure legend, table legend, main text, or Methods section.

n/a	Confirmed	
☐	☒	The exact sample size (*n*) for each experimental group/condition, given as a discrete number and unit of measurement
☐	☒	A statement on whether measurements were taken from distinct samples or whether the same sample was measured repeatedly
☐	☒	The statistical test(s) used AND whether they are one- or two-sided<br>*Only common tests should be described solely by name; describe more complex techniques in the Methods section.*
☒	☐	A description of all covariates tested
☐	☒	A description of any assumptions or corrections, such as tests of normality and adjustment for multiple comparisons
☐	☒	A full description of the statistical parameters including central tendency (e.g. means) or other basic estimates (e.g. regression coefficient) AND variation (e.g. standard deviation) or associated estimates of uncertainty (e.g. confidence intervals)
☐	☒	For null hypothesis testing, the test statistic (e.g. *F*, *t*, *r*) with confidence intervals, effect sizes, degrees of freedom and *P* value noted<br>*Give P values as exact values whenever suitable.*
☒	☐	For Bayesian analysis, information on the choice of priors and Markov chain Monte Carlo settings
☒	☐	For hierarchical and complex designs, identification of the appropriate level for tests and full reporting of outcomes
☐	☒	Estimates of effect sizes (e.g. Cohen's *d*, Pearson's *r*), indicating how they were calculated

Our web collection on statistics for biologists contains articles on many of the points above.

Software and code

Policy information about availability of computer code

Data collection	BD LSRII (IMED) flow cytometer was used for analyzing flow cytometry data. CFX96 Touch Real-Time PCR Detection System (Bio-Rad) was used for qRT-PCR. Olympus IX-51 microscope was used for image acquire and analysis. Next-generation sequencing data was collected by HiSeq 3000 or NovaSeq SP.
Data analysis	The following software was used for data analysis: Hisat(v2.2.0), Subread2(v2.0.3) and Deseq2(v1.32.0) were used for Bulk RNAseq analysis. Bowtie2(v2.4.2), MACS2(v2.2.8), Deeptools(v2.0), and ChromHMM(v1.24) were used for ChIP-seq analysis. GraphPad Prsim(v9.4.0) was used for statistical analysis. FlowJo(v10) was used for flow cytometry data analysis.

For manuscripts utilizing custom algorithms or software that are central to the research but not yet described in published literature, software must be made available to editors and reviewers. We strongly encourage code deposition in a community repository (e.g. GitHub). See the Nature Portfolio guidelines for submitting code & software for further information.

Data

Policy information about availability of data

All manuscripts must include a data availability statement. This statement should provide the following information, where applicable:

- Accession codes, unique identifiers, or web links for publicly available datasets
- A description of any restrictions on data availability
- For clinical datasets or third party data, please ensure that the statement adheres to our policy

The raw data of RNA-seq, ChIP-seq, and whole exome sequencing (WES) have been deposited at the Gene Expression Omnibus (GEO) under the accession number GEO: GSE237454, GSE234640, and GSE234824, respectively. All processed data are available within the Article, the Supplementary Information, and the Source Data file. Source data are provided in this paper.

Research involving human participants, their data, or biological material

Policy information about studies with human participants or human data. See also policy information about sex, gender (identity/presentation), and sexual orientation and race, ethnicity and racism.

Reporting on sex and gender	No human research participants were involved in this project.
Reporting on race, ethnicity, or other socially relevant groupings	N/A
Population characteristics	N/A
Recruitment	N/A
Ethics oversight	N/A

Note that full information on the approval of the study protocol must also be provided in the manuscript.

Field-specific reporting

Please select the one below that is the best fit for your research. If you are not sure, read the appropriate sections before making your selection.

☒ Life sciences ☐ Behavioural & social sciences ☐ Ecological, evolutionary & environmental sciences

For a reference copy of the document with all sections, see nature.com/documents/nr-reporting-summary-flat.pdf

Life sciences study design

All studies must disclose on these points even when the disclosure is negative.

Sample size	No statistical method was used to predetermine sample size. The sample sizes were determined based on our previous studies and the experience of the authors. We expect changes in genes expression and function measurements to be detected with n=3 samples per group unless otherwise noted.
Data exclusions	No data were excluded from analysis.
Replication	All in vitro studies were replicated three times independently, and in vivo studies were done two times independently. Replication details for each assay were provided in the manuscript.
Randomization	For in vivo experiments, animals were randomly assigned into different treatment groups before initiating the experiments. For in vitro experiments, all samples were analyzed equally with no subsampling.
Blinding	Tumor measurements and body weight collection were not blinded due to staffing shortage, but further histological analysis and IHC and IF images were acquired and analyzed in a blinded fashion. For other experiments, blinding was not applicable because the researchers themselves designed, performed, and analyzed the experiments.

Reporting for specific materials, systems and methods

We require information from authors about some types of materials, experimental systems and methods used in many studies. Here, indicate whether each material, system or method listed is relevant to your study. If you are not sure if a list item applies to your research, read the appropriate section before selecting a response.

Materials & experimental systems

n/a	Involved in the study
☐	☒ Antibodies
☐	☒ Eukaryotic cell lines
☒	☐ Palaeontology and archaeology
☐	☒ Animals and other organisms
☒	☐ Clinical data
☒	☐ Dual use research of concern
☒	☐ Plants

Methods

n/a	Involved in the study
☐	☒ ChIP-seq
☐	☒ Flow cytometry
☒	☐ MRI-based neuroimaging

Antibodies

Antibodies used

The following antibodies were used in the study (Supplier; Catalog number; Clone name/Lot; Dilution):
1. Anti-KMT2D (Sigma-Aldrich, ABE1867, 9Q013R, 1:500 for WB, 1:50 for IHC, 1:100 for ChIP)
2. Anti-LDHB (Santa Cruz Biotechnology, sc-100775, 431.1, 1:1000 for WB)
3. Anti-LDHB (Proteintech, 14824-1-AP, AG6605, 1:200 for IHC)
4. Anti-PGK1 (Santa Cruz Biotechnology, sc-130335, 14, 1:1000 for WB, 1:100 for IHC)
5. Anti-H3K4me1 (Abcam, ab8895, 1:10000 for WB, 1:100 for ChIP)
6. Anti-H3K27ac (Active Motif, 39133, 1:100 for ChIP)
7. Anti-H3K4me3 (Abcam, 8580, 1:100 for ChIP)
8. Anti-H3K9me3 (Abcam, 8898, 1:100 for ChIP)
9. Anti-H3K27me3 (Abcam, 6002, 1:100 for ChIP)
10. Anti-H3(Abcam, ab1791, 1:10000 for WB, 1:100 for ChIP)
11. Anti-α-tubulin (Sigma-Aldrich, T5168, B-5-1-2, 1:10000 for WB)
12. Anti-Cleaved Caspase-3 (Cell Signaling Technology, 9661, 1:1000 for WB)
13. Anti-Cleaved PARP (Cell Signaling Technology, 5625, 1:1000 for WB)
14. Anti-FANCD2 (Abcam, ab108928, 1:2000 for WB)
15. Anti-FANCI (Abcam, ab245219, 1:5000 for WB)
16. Anti-FANCG (Thermo Fisher Scientific, 10215-1-AP, 1:1000 for WB)
17. Anti-FANCL (Proteintech, 66639-1-Ig, 6E2B6, 1:1000 for WB)
18. Anti-KMT2A (Novus Biologicals, NB600-248, 1:1000 for WB)
19. Anti-KMT2B (Cell Signaling Technology, 47097, 1:1000 for WB)
20. Anti-KMT2C (Sigma-Aldrich, SAB1300082, 1:1000 for WB)
21. Anti-BrdU (Abcam, ab6326, 1:100 for IF)
22. Anti-Phospho-AMPKα (Cell Signaling Technology, 2535, 40H9, 1:1000 for WB)
23. Anti-Pan cytokeratin (Abcam, ab9377, 1:50 for IHC)
24. Anti-Pan cytokeratin (Novus Biologicals, NBP2-29429, 1:50 for IF)
25. Anti-Ki-67 (Abcam, ab15580, 1:100 for IF)

Validation

All commercial antibodies were validated by the manufacturers as indicated on their websites. Antibodies used for western blotting were further validated by the molecular weights of the target proteins. The validation statements of all antibodies are publicly accessible on the manufacturers' websites:
1. https://www.sigmaaldrich.com/US/en/product/mm/abe1867
2. https://www.scbt.com/p/ldh-b-antibody-431-1
3. https://www.ptglab.com/products/LDHB-Antibody-14824-1-AP.htm
4. https://www.scbt.com/p/pgk1-antibody-14
5. https://www.abcam.com/products/primary-antibodies/histone-h3-mono-methyl-k4-antibody-chip-grade-ab8895.html
6. https://www.activemotif.com/catalog/details/39133/histone-h3-acetyl-lys27-antibody-pab
7. https://www.abcam.com/products/primary-antibodies/histone-h3-tri-methyl-k4-antibody-chip-grade-ab8580.html
8. https://www.abcam.com/products/primary-antibodies/histone-h3-tri-methyl-k9-antibody-chip-grade-ab8898.html
9. https://www.abcam.com/products/primary-antibodies/histone-h3-tri-methyl-k27-antibody-mabcam-6002-chip-grade-ab6002.html
10. https://www.abcam.com/products/primary-antibodies/histone-h3-antibody-nuclear-marker-and-chip-grade-ab1791.html
11. https://www.sigmaaldrich.com/US/en/product/sigma/t5168
12. https://www.cellsignal.com/products/primary-antibodies/cleaved-caspase-3-asp175-antibody/9661
13. https://www.cellsignal.com/products/primary-antibodies/cleaved-parp-asp214-d64e10-xp-174-rabbit-mab/5625
14. https://www.abcam.com/products/primary-antibodies/fancd2-antibody-epr2302-ab108928.html
15. https://www.abcam.com/products/primary-antibodies/fanci-antibody-epr23903-124-ab245219.html
16. https://www.thermofisher.com/antibody/product/FANCG-Antibody-Polyclonal/10215-1-AP
17. https://www.ptglab.com/products/FANCL-Antibody-66639-1-Ig.htm
18. https://www.novusbio.com/products/kmt2a-mll-antibody_nb600-248
19. https://www.cellsignal.com/products/primary-antibodies/mll2-kmt2b-e3m1v-rabbit-mab-amino-terminal-antigen/47097
20. https://www.sigmaaldrich.com/US/en/product/sigma/sab1300082
21. https://www.abcam.com/products/primary-antibodies/brdu-antibody-bu175-icr1-proliferation-marker-ab6326.html
22. https://www.cellsignal.com/products/primary-antibodies/phospho-ampka-thr172-40h9-rabbit-mab/2535
23. https://www.abcam.com/products/primary-antibodies/wide-spectrum-cytokeratin-antibody-ab9377.html
24. https://www.novusbio.com/products/cytokeratin-pan-antibody-ae-1-ae-3_nbp2-29429
25. https://www.abcam.com/products/primary-antibodies/ki67-antibody-ab15580.html

Eukaryotic cell lines

Policy information about cell lines and Sex and Gender in Research

Cell line source(s)	293T/17 was purchased from ATCC (ATCC, Cat#CRL-11268). SCC23, SCC1 and SCC9 all were originally from Dr. Thomas E. Carey at University of Michigan at Ann Arbor. SCC46 and SCC74A were from Drs. Zhong Chen and Carter Van Waes at National Institute on Deafness and Other Communication disorders, National Institute of Health with the permission from Dr. Thomas E. Carey.
Authentication	The cell lines were not authenticated.
Mycoplasma contamination	All cell lines tested negative for mycoplasma contamination.
Commonly misidentified lines (See ICLAC register)	No commonly misidentified cell lines were used in this study.

Animals and other research organisms

Policy information about studies involving animals; ARRIVE guidelines recommended for reporting animal research, and Sex and Gender in Research

Laboratory animals	Kmt2df/f (JAX:032152) mice, K14CreER (JAX:005107) mice, nude mice (JAX:002019), and NSG mice (JAX:005557) were purchased from the Jackson Laboratory. K14CreER mice were backcrossed to C57BL/6J for five generations and then were crossed with Kmt2df/f mice to generate K14CreER;Kmt2df/+ mice. For induction of HNSCC, six-week-old mice were treated with 40 μg/mL 4NQO-containing drinking water for 16 weeks and then normal drinking water for another 9-10 weeks for tumor formation. six-week-old female nude mice were used for the orthotopic HNSCC injection, and six-week-old female NSG mice were used for human HNSCC PDX xenografts.
Wild animals	This project did not use wild animals.
Reporting on sex	Both sexes as male and female littermate mice were used and divided into different experimental groups for 4NOQ HNSCC model. For nude mice and NSG mice, we used females.
Field-collected samples	No field samples collected.
Ethics oversight	Animal use was approved by UCLA Animal Research Committee (Protocol Number#ARC-2007-062).

Note that full information on the approval of the study protocol must also be provided in the manuscript.

Plants

Seed stocks	The study did not involve plants.
Novel plant genotypes	The study did not involve plants.
Authentication	The study did not involve plants.

ChIP-seq

Data deposition

☒ Confirm that both raw and final processed data have been deposited in a public database such as GEO.

☒ Confirm that you have deposited or provided access to graph files (e.g. BED files) for the called peaks.

Data access links *May remain private before publication.*	The ChIP-seq data is available in GEO: GSE234640.
Files in database submission	14 files.
Genome browser session (e.g. UCSC)	We will provide the line upon request and will be added to the manuscript prior to acceptance.

Methodology

Replicates	1
Sequencing depth	For each library, about 5G bases were sequenced with 50-bp single ends reads. The Uniquely mapped reads for each library is above 85%.
Antibodies	anti-KMT2D (Millipore Sigma, Cat#ABE1867), anti-H3K4me1 (Abcam, Cat#ab8895), anti-H3K27ac (Active Motif, Cat#39133), anti-H3K4me3 (Abcam, Cat#ab8580), anti-H3K9me3 (Abcam, Cat#ab8898), anti-H3K27me3 (Abcam, Cat#ab6002), and anti-H3 (Abcam, Cat#ab1791).
Peak calling parameters	Reads are mapped to hg19 by bowtie2(2.4.2), then uniquely mapped reads were used for peak calling by MACS2 (2.2.8) with parameter -g hs -B -f BAM --p 0.01
Data quality	For KMT2D, 13808 peaks were identified;<br>For H3K4me1, 63782 peaks were identified;<br>For H3K27ac, 63701 peaks were identified;<br>For H3K4me3, 13851 peaks were identified;<br>For H3K9me3, 28032 peaks were identified;<br>For H3K27me3, 71812 peaks were identified.
Software	Bowtie2(2.4.2), MACS2(2.2.8), deeptools(2.0)

Flow Cytometry

Plots

Confirm that:

☒ The axis labels state the marker and fluorochrome used (e.g. CD4-FITC).

☒ The axis scales are clearly visible. Include numbers along axes only for bottom left plot of group (a 'group' is an analysis of identical markers).

☒ All plots are contour plots with outliers or pseudocolor plots.

☒ A numerical value for number of cells or percentage (with statistics) is provided.

Methodology

Sample preparation	For sorting of PX458 transfected HNSCC cells, cells were transfected with indicated plasmids for 24 hours, and then GFP-positive cells were harvested for further experiments.<br>Flow cytometry apoptosis analysis was performed on KMT2D-KO and KMT2D-WT SCC23 cells that were subjected to glucose deprivation or treated with 2-DG and/or low-dose MMC. The apoptotic rates were measured using FITC Annexin V Apoptosis Detection Kit (BD Biosciences, Cat#556547) according to the manufacturer’s protocol.
Instrument	LSRII (IMED) flow cytometer from BD Biosciences.
Software	FlowJo v10.
Cell population abundance	At least 3000 GFP+ cells were collected from PX458-sgKMT2D-transfected HNSCC cells for further cell culture.
Gating strategy	FSC-A/SSC-A gates of the starting cell population were used for identifying events corresponding to cells, and then FSC-H/SSC-A gates were used for the singlets. The single population was further analyzed for GFP or FITC-Annexin V and PI.

☒ Tick this box to confirm that a figure exemplifying the gating strategy is provided in the Supplementary Information.
